# Research on security risk assessment mechanism of important event based on multi-source data

**DOI:** 10.1038/s41598-022-08079-2

**Published:** 2022-03-08

**Authors:** Qilei Wang

**Affiliations:** China People’s Police University, Research Centre for Modern Police Technology and Equipment, Langfang, 065000 Hebei China

**Keywords:** Risk factors, Computer science

## Abstract

In order to effectively assess all types of security risks in the important event, the important decision-making basis for security risk warning and emergency management of important event is provided by analyzing the coupling relationship and evolution mechanism between various risks. The criminal causes, management defects, security and emergency system construction are analyzed from the possibility of accidents and risks. The multi-source data risk assessment system based on five subsystems and its index set of human factors, management factors, site factors, event factors and audit factors are proposed. The weight of each index in the assessment system is determined by the method of information entropy, and then the risk grade of important event is determined according to the weight calculation function and the improved fuzzy matter element model. The verification with an example shows that: the risk assessment model is optimized by combining entropy weight with fuzzy matter-element model, the influence of weight data extreme value was weakened, the qualitative description and quantitative analysis of multi-source data could be combined, and the subjective error was reduced. The risk grade of important event is reasonably evaluated, and the assessment effect is basically consistent with the expert inspection analysis, which shows that the method had certain application value.

## Introduction

With the change of international and domestic forms, the important event involving political, economic, cultural, sports, entertainment and other industries have been increasing, such as G20 summits, APEC conferences, automobile exhibitions, football and other large-scale sports events, star concerts, etc. The political and cultural exchanges are promoted, and economic development also is promoted to a certain extent due to the holding of these important events^1,2^. However, the hidden dangers about how can it be resolved in all kinds of important event to become a priority issue for the organizational unit. The crowded stampede, fire and explosion, public security crimes, terrorist attacks, mass disturbances and a series of security issues are often happened due to the large number of people and the complex types involved^3,4^. Such as the hundreds of terrorist attacks are happened before and after the 2008 Beijing Olympic Games, In 2014, hundreds of people are killed and injured in a stampede on the Shanghai Bund, and the 2018 Venezuelan national celebration is attacked by drone explosive and so on. The lack of security risk prevention and emergency response capacity for the incidents are reflect from some extent. Through analysis, once the security issue was occurred in the process of organizing activities, the emergencies are difficult to be disposed due to the large number of personnel. In addition to causing casualties, equipment damage and other serious social hazards such as political and economic. Therefore, the organization of event are analyzed, all kinds of security risks are study, and the good job of risk assessment in advance is done, which has become a research hotspot for many event organization departments and scholars^5^. The security of important event is affected by many factors, and there are still a certain relationship between the factors. The quantitative and qualitative factors are fully consider by analyzed multi-source data. The security influencing factors and security risk index system of important event are established, which played an important role in the safety risk assessment mechanism of important event. For example, Zhang^6^ discussed the important influencing factors and significance of risk assessment for the security of important event, which find that security indicators can be effectively improved by the risk assessment. The possibility of terrorist attack is analyzed in subway and the specific index system of risk assessment is established by Matsika^7^. The many methods are used in security risk assessment at present, which mainly focused on using various mathematical models to analyze the multi-source data of the problems faced and obtain the safety risk indicators, such as fuzzy theory, entropy weight method, analytic hierarchy process and mutation theory. Svalin^2^ give the effectiveness of risk assessment and early warning of police force in emergency police cases from the point of view of risk prediction system construction, model construction and data acquisition. The method of neural network and expert reasoning is put forward to quantitatively analyze the risk factors affecting the security prevention system and improve the accuracy of the assessment model^8^. Shamala^9^ study the impact of information acquisition quality on safety risk assessment and how to effectively manage risk. Shen^10^ presented a novel approach for assessing the potential risk of serious crime events (e.g. terrorist attack), then analyze and prove its validity. However, the risk assessment of important event mainly focus on fire risk and food safety, which included static and dynamic risk, human and equipment factors. The research on security risk of specific activities was relatively few, and the assessment and detection index screening of human, object and equipment were vulnerable to subjective influence. The weighted quantitative analysis also depends on the professional knowledge level of the evaluator, and the judgment matrix standard is revised with strong subjectivity and lack of scientific theory and method^11–13^.

The superiority of combining entropy weight and fuzzy matter element in objective evaluation. In this paper, the dynamic model of public security risk assessment for important event is established by using entropy weight and fuzzy matter element model based on the relevant national standards, expert experience and field assessment. Then the multi-source data such as human factors, management factors, site equipment factors, event factors and audit factors are respectively empowering, the subjective factors are reduced and objective calculation accuracy is increased. The security risk index and the possible problems of the event are analyzed by taking the certain event as a column, and the preventive measures are put forward which is of great practical significance to the security risk assessment of the important event.

## Risk assessment

### Risk indicators

The security risk of important event is huge, which easy to be affected by various factors, and the relationship between the factors have certain coupling characteristics. The mutual influence between the factors are caused due to a certain factor changes, which lead to public security emergencies and easy to be uncontrolled^14–16^. Therefore, effective classification of influencing factors are of great significance. This paper summarize the influencing factors into five aspects: human, management, site equipment, events, related audit, and established four levels of assessment index system based on the possibility of security risk, security management defects and the construction of emergency security disposal system. The requirements of the standard are not only necessary to be met, but an index system to objectively reflect the composition and internal relationship of various factors in security assessment need to be established in line with the precise, comprehensive and practical criteria. And each assessment index can be divided into an orderly level to make it orderly, the availability of assessment index data and the maximum practical value of risk assessment mechanism are considered simultaneously.

Human factors A mainly caused by human factors risk indicators, select the composition of participants, important people, organizers, event management personnel ability as the main dimension indicators, so as to improve the composition of personnel and business level, improve the ability of personnel. According to the assessment results, the composition of personnel and the level of personnel operational capacity are improved.

The management and prevention factors B are the basic guarantee for the security operation of important events. The relevant schemes and technical means can be optimized by selecting the dimensions of plan formulation, emergency management, inspection and examination, technical and tool defense, specific execution.

The site and equipment factors C are the basic requirement for the security holding of important event, the site situation, the condition of the goods and the condition of the equipment are selected as the main assessment index. The site selection, the daily management and maintenance of the equipment can be effectively carried out according to the situation, and the old equipment can be optimized regularly.

Event risk factors D are an important trigger point of activity security risk, which mainly involve the dimensions of domestic and foreign situation, type and nature of event, social situation and attention of event. The risk can be reduced by social guidance and prevention according to assessment report.

Relevant audit factors E are the permitted basic condition of event, which mainly carry out by various qualification audits are formed from approval organs and organizational organs, event site maintenance and security, emergency disposal audit and other dimensions. The initial conditions for security risks are constituted by the elements.

**Figure 1:**
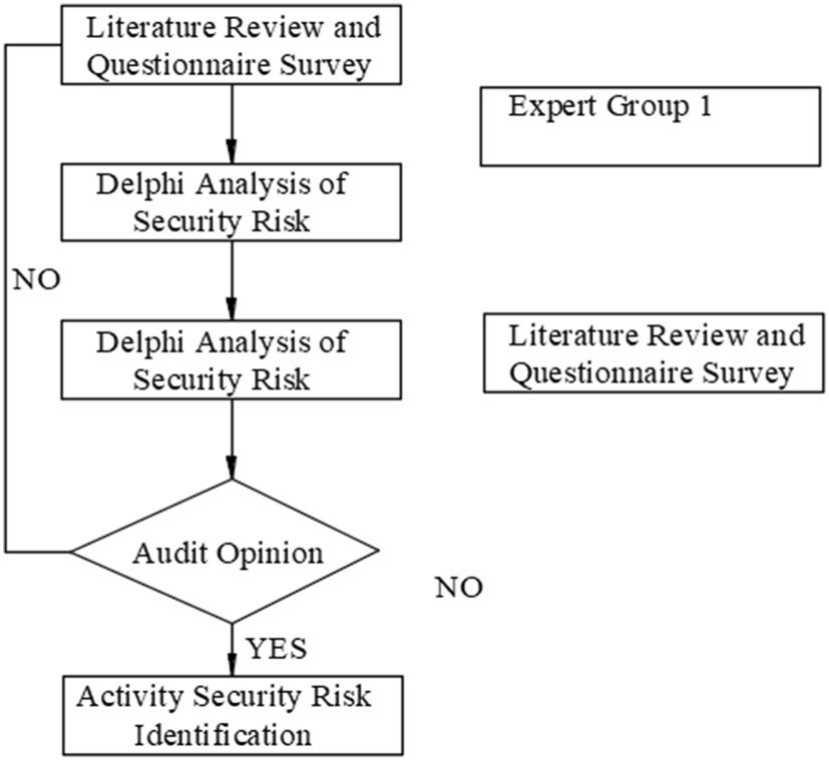
Risk factors analysis process.

The risk element composition analysis process is shown in Fig. 1.

The achievements of relevant scholars in the research of security risk assessment are drawn on the basis of comprehensive analysis and investigation, and multi-layer factors set index are established according to the relevant security information of events and solicit expert suggestions, which included 4 level index and 63 basic influencing factors, as shown in Table 1.

**Table 1:** Security risk assessment indicators system of important event.

I level	II level	III level	V level
Security risks indicators of important event	Human factors A	Composition of participants A_1_	Age composition A_11_
			Education background A_12_
			Religious belief A_13_
			Nationality A_14_
			State of mind A_15_
			Registration data review A_16_
		Important persons A_2_	Domestic and foreign leaders A_21_
			Famous man A_22_
			Sensitive personnel A_23_
		Organizers A_3_	Experience A_31_
			Capacity A_32_
			Service provider A_33_
		Event managers A_4_	Professional qualityA_41_
			Manning A_42_
			Background information A_43_
	Management and prevention factors B	Plan formulation B_1_	Evacuation plan B_11_
			Emergency response plan B_12_
			Prevention plan B_13_
			Plan Implementation Progress B_14_
		Basic management B_2_	Evacuation equipment B_21_
			Security equipment B_22_
			Personnel quality B_23_
			Management system B_24_
			Medical care personnel B_25_
		Inspection and examination B_3_	Inspection review systemB_31_
			Professional quality and attitude B_32_
			Supervision system B_33_
		Technical and tool defense B_4_	Camera security system B_41_
			Face recognition systemB_42_
			Distribution of security organs B_43_
		Specific execution B_5_	Event program implementation B_51_
			Field equipment operation B_52_
	Site and equipment factors C	Site conditions C_1_	Site construction C_11_
			Site experience C_12_
			Road traffic around the site C_13_
		Item condition C_2_	Food safety situation C_21_
			Gas placement C_22_
			Circuit distribution C_23_
			Precious and forbidden items C_24_
		Equipment situation C_3_	General equipment C_31_
			Operating equipment C_32_
		Weather situation C_4_	Thunderbolt C_41_
			Elements C_42_
			Temperature C_43_
	Event factors D	Domestic and international situation D_1_	Foreign terror D_11_
			Domestic terrorists D_12_
			Crime rate D_13_
			Contradiction D_14_
		Event type and nature D_2_	Event Influence D_21_
			Event interactivity D_22_
			All types of attention D_23_
		Event social situation D_3_	Public opinion information D_31_
			Political religious sensitivity D_32_
			Ticket issuance D_33_
	Audit factors E	Approval and organization organs E_1_	Approval document review E_11_
			Signing agreement review E_12_
		Event site review E_2_	Site related condition audit E_21_
			Temporary construction facility audit E_22_
		Security and safety precautions E_3_	Security company qualification E_31_
			Attention of government departments E_32_
		Examination and verification of qualification E_4_	Quality audit of activity party E_41_
			Catering qualification audit E_42_

### Interactions of risk

**Figure 2:**
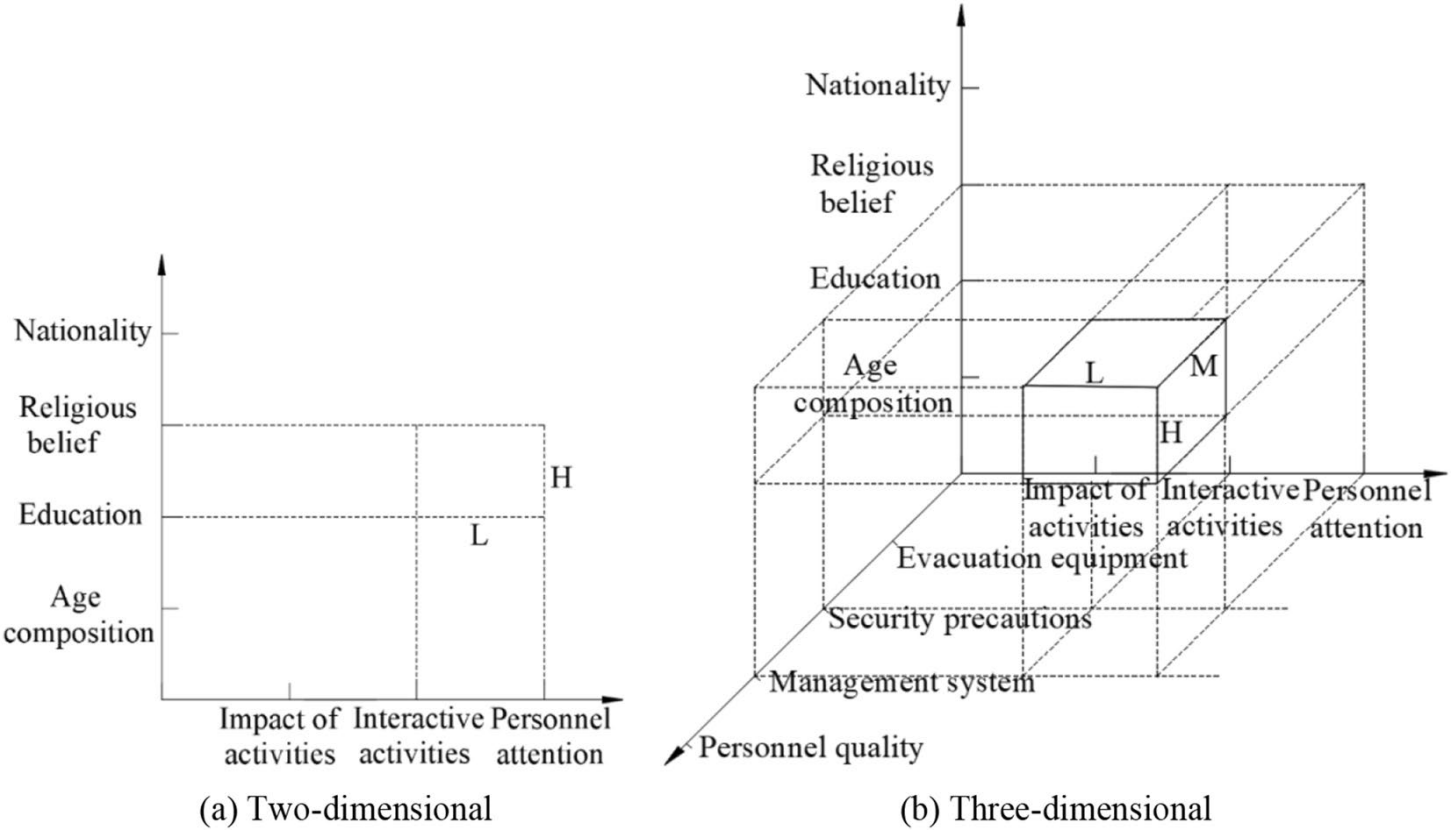
Risk scenario coupling relationship.

The subfactors contained under a single, two or three main factors are staggered analyzed, the risk can be further identified to obtain the risk sources from different perspectives. For example, two-dimensional A_1_&D_2_ risk scenarios as shown in Fig. 2a, The new perspective of risk is created by the interaction between two subfactors of cultural education and event interactivity, That is, the cultural level and interaction of the participants are different requirements due to different scenarios and different content of important event. The serious consequences are easy to be caused if the two match improperly. Similarly, for a three-dimensional A_1_&D_2_&B_2_ risk scenarios as shown in Fig. 2b.The new three-dimensional perspective of risk is taken shape by the interaction between three sub-factors of cultural education, management system and event interactivity. For example, the interactions are different between different of interaction ways, academic qualifications and management methods, which required managers to conduct interaction and management training according to the actual personnel culture. Otherwise, some avoidable risks will be happen in the course of the activity.

According to this method, the risks caused by the interaction of multiple subfactors are identified. In addition, the side length of the geometry generated by the interaction can be measured by the probability or consequence of each subfactor in the coordinate system shown in Fig. 2. The area or volume of the resulting geometric can be used to measure the risk degree under the interaction of various factors in a risk scenario. Taking the probability measure as an example, when the dimension of the risk scenario are less than or equal to 3, we can obtain:

1 $$M_{risk} = m_{x} m_{y} m_{z}$$

*M*_risk_ is defined as the risk degree under the interaction of three factors in the risk scenario, *m*_x_, *m*_y_, *m*_z_ represent the side length of the geometry generated by the interaction in the risk coordinate system, that is, the posterior probability of a single risk factor. Specifically, *m*_z_ = 1 when the *M*_risk_ is the two-dimensional risk scenario.

## Entropy weight and model

In the event of danger of a major event, the chain reaction of many factors are caused, which will lead to more serious problems are caused due to the superposition of each other. The multiple problems need to be considered at the same time when the risk grade assessment is considered, the judgment matrix with fuzzy consistency is select for analyze the multi-objective decision.

### Index weight entropy weight

Entropy weight method is an objective assignment method, which mainly use the utility value of information to determine the index weight. The effective use of entropy weight function model to evaluate the security risk of important event is mainly used by the factors of affect the security of important event are effective differentiated and ranked, and the entropy value is determined. The calculation and analysis are carried out by comprehensive use of the order of the information obtained. The weight value of assessment index is mainly used as the initial calculation matrix $X = \left\{ {X_{ij} } \right\}_{m \times n}$, *m* is the number of assessment object, the *n* is the number of assessment indexes. In order to reduce the difference of dimension, order of magnitude and quality of data index, the selected data index are normalized:

2 $$y_{ij} = \frac{{x_{ij} }}{{\sum\nolimits_{i = 1}^{m} {x_{ij} } }}, \quad i = 1,2, \ldots ,m; \quad j = 1,2, \ldots ,n$$

According to the analysis,$0 \le y_{ij} \le 1$, the normalized data matrix can be obtained after calculation:

3 $$Y = \left( {Y_{ij} } \right)_{m \times n} , \; \; \; i = 1,2, \ldots ,m; \; \; j = 1,2, \ldots ,n$$

Clearly, according to the above formula, the entropy of item *j* is:

4 $$e_{j} = - k\sum\limits_{i = 1}^{m} {y_{ij} \ln y_{ij} }, \; \; \; j = 1,2, \ldots ,n$$

Among them, the constant *k* is 1/*lnm*, which is mainly related to the number of assessment objects. The efficiency value *e*_j_ of the *j* index entropy value are determined. When the order degree of the system is higher, the *e*_j_ value tends to 0, which will result in a higher value for comprehensive assessment. When order degree of the system is lower, the *e*_*j*_ value tends to 1, which will result in a lower value for comprehensive assessment.

Therefore, the index utility value are effectively determined by the difference *h*_*j*_ between index entropy *e*_*j*_ and 1 in the assessment system, that is:

5 $$h_{j} = 1 - e_{j},\;\;j = 1,2, \ldots,n$$

As the core of entropy method, the information utility value have important influence on the importance of assessment result. The weight of each index is estimated based on the characteristics of entropy method, and the weight of item j can be obtained after analysis:

6 $$w_{j} = \frac{{h_{j} }}{{\sum\nolimits_{j = 1}^{n} {h_{j} } }},\quad j = 1,2, \ldots ,n$$

### Construction of fuzzy element model

In order to effectively distinguish between factors, the assessment model based on the ordered triplet R (*M*, *c*, *v*) are used as the basic element to describe things to construct fuzzy elements in the multi-objective decision-making system. Where *M* is the assessment object, the *c* is the M characteristic index, and the v is the fuzzy value. The assessment object M has *n* characteristics $c_{1} ,c_{2} , \ldots ,c_{n}$ and corresponding fuzzy values $v_{1} ,v_{2} , \ldots ,v_{n}$.Suppose *R* as the *n* dimension fuzzy object element, when there are *m* assessment objects, each object has the n dimension fuzzy value element. The *n* dimensional fuzzy object element of *m* object are compounded by the mixed expression forms, expressed as *R*_*mn*_:

7 $$R_{mn} = \left( {\begin{array}{*{20}c} {} & {M_{1} } & {M_{2} } & \cdots & {M_{m} } \\ {c_{1} } & {X_{11} } & {X_{21} } & \cdots & {X_{m1} } \\ {c_{2} } & {X_{12} } & {X_{22} } & \cdots & {X_{m2} } \\ \cdots & \cdots & \cdots & \cdots & \cdots \\ {c_{n} } & {X_{1n} } & {X_{2n} } & \cdots & {X_{mn} } \\ \end{array} } \right)$$

where *M*_*i*_ is the *i* objec*t*, $i = 1,2, \ldots ,m$; *c *_*j*_ is the *j* item characteristics, $j = 1,2, \ldots ,n$; *X*_*ij*_ is the fuzzy value corresponding to the *j* item characteristics of the *i* object, that is, membership degree.

The fuzzy value corresponding to each assessment index is determined, and the subordinate relation of each index is important to the influence degree of the assessment index. Because the eigenvalues of each index dimension are different, and the eigenvalue difference have certain influence on the assessment result. Comparative study showed that the eigenvalue of each assessment index have an important influence on the good rate, but the trend is different due to determined by the nature of index. In order to clarify the relationship between the good properties of each index and the membership degree, the following formulas are adopted:

8 $$\text{Index}\; \text{of}\; \text{the} \; \text{bigger} \; \text{the}\; \text{better}{:} \; \mu_{ij} = \frac{{X_{ij} }}{{\max X_{ij} }}$$

9 $$\text{Index} \; \text{of} \;\text{the} \;\text{smaller} \;\text{the} \;\text{better}{:}\; \mu_{ij} = \frac{{\min X_{ij} }}{{X_{ij} }}$$

Equations (8) and (9) are different trend indexes respectively. The two formulas are related to each other, but do not affect each other, because they are aimed at different objects. The composite entropy fuzzy matter are calculated based on the membership degree relation of the fuzzy value *X*_*ij*_ of *n* dimension fuzzy matter matter of *m* object. According to the principle of superior subordinate degree, the corresponding composite elements are obtained:

10 $$\tilde{R}_{mn} = \left( {\begin{array}{*{20}c} {} & {M_{1} } & {M_{2} } & \cdots & {M_{m} } \\ {c_{1} } & {\mu_{11} } & {\mu_{21} } & \cdots & {\mu_{m1} } \\ {c_{2} } & {\mu_{12} } & {\mu_{22} } & \cdots & {\mu_{m2} } \\ \cdots & \cdots & \cdots & \cdots & \cdots \\ {c_{n} } & {\mu_{1n} } & {\mu_{2n} } & \cdots & {\mu_{mn} } \\ \end{array} } \right)$$

### Closeness-degree calculation

As an important basis to measure the security risk assessment index of major activities, the difference between the assessment object and the assessment standard value are determined by the size of matched value. Combined with the index data to analyze and calculate the closeness of the assessment object based on Euclid closeness-degree mathematical model *ρH*_*j*_. According to the results of the calculation, the order of the advantages and disadvantages of multiple assessment objects are carried out, and the calculating formula for the closeness-degree of compound fuzzy elements is constructed:

11 $$R_{\rho H} = \left[ {\begin{array}{*{20}c} {} & {M_{1} } & {M_{2} } & \cdots & {M_{m} } \\ {\rho H_{j} } & {\rho H_{1} } & {\rho H_{2} } & \cdots & {\rho H_{m} } \\ \end{array} } \right]$$

where M is the assessment object, $\rho H_{j}$ is the closeness-degree. The computational formula of the *j* closeness-degree in fuzzy element matrix R_*ρH*_ as follows:

12 $$\rho H_{j} = 1 - \sqrt {\sum\limits_{i = 1}^{n} {w_{i} \mu_{ij} } } , \quad (j = 1,2, \ldots ,m)$$

The entropy weight value and standard value of each assessment index of security risk assessment of important events are compared and analyzed. The formula as follows:

13 $$Z_{j} = \sum\limits_{i = 1}^{n} {w_{i} \times b_{ij} }$$

where *Z*_*j*_ is the combined assessment values for *j* assessment subjects.

## Assessment example building

In order to verify the above risk assessment model, the different events are selected to assessment comparative for effectively evaluated the security risk of different important events and analyzed the security risk assessment indexes of different important events. According to the index system, two kinds of events are selected for comprehensive assessment. The security risk status of different events are sorted, and the cause analysis is carried out.

### The assessment objects

Several large-scale events held in recent years are selected as the assessment objects based on 63 assessment indicators in 5 aspects: human, management, site, event and audit. Two music festivals in Kunming (P1, 2018) and Zhangbei Grassland (P2, 2019), and two large-scale football matches in Beijing (P3, 2019) and Guangzhou (P4, 2019) are selected as assessment objects respectively.

### Risk comparative analysis

Combined with fuzzy matter element model and entropy weight calculation weight method, the above four events are used as cases for security risk assessment. According to the combination of quantitative and qualitative, the specific measured value of major events are taken as the quantitative index value, and the weighted average of the expert assessment group combined with the data scoring are taken as the qualitative index value. After the assessment is completed, the assessment index value is normalized by the judgment matrix formula (8) or formula (9). After the processing is completed, the entropy value and entropy weight of the assessment object are processed according to formula (4) and formula (6). Then the calculated value is imported into formula (9) to obtain the functional membership degree of the corresponding index. The calculation results of entropy value and entropy weight are shown in Table 2.

**Table 2:** Evaluation index entropy (*H*_j_) and entropy weight(*w*_*j*_).

Index	P_1_		P_2_		P_3_		P_4_	
	*H*_*j*_	* w*_*j*_	*H*_*j*_	* w*_*j*_	*H*_*j*_	* w*_*j*_	*H*_*j*_	* w*_*j*_
A_11_	0.35	0.0264	0.33	0.0252	0.29	0.0239	0.28	0.0227
A_12_	0.201	0.0191	0.213	0.0197	0.232	0.0209	0.243	0.0204
A_13_	0.211	0.0197	0.254	0.0206	0.255	0.0208	0.265	0.0216
A_14_	0.187	0.0167	0.197	0.0177	0.198	0.0181	0.201	0.0184
A_15_	0.241	0.0206	0.235	0.0184	0.287	0.0212	0.294	0.0244
A_16_	0.313	0.0233	0.331	0.0237	0.367	0.0271	0.376	0.0276
A_21_	0.334	0.0247	0.314	0.0232	0.312	0.0219	0.332	0.0244
A_22_	0.365	0.0269	0.353	0.0257	0.321	0.0224	0.335	0.0228
A_23_	0.341	0.0247	0.337	0.0236	0.297	0.0216	0.307	0.0224
A_31_	0.187	0.0137	0.179	0.0128	0.199	0.0141	0.188	0.0132
A_32_	0.195	0.0146	0.181	0.0136	0.201	0.0142	0.194	0.0141
A_33_	0.189	0.0142	0.184	0.0132	0.211	0.0146	0.204	0.0144
A_41_	0.333	0.0246	0.339	0.0244	0.303	0.0224	0.323	0.0241
A_42_	0.312	0.0232	0.321	0.0236	0.312	0.0227	0.304	0.0223
A_43_	0.342	0.0249	0.335	0.0245	0.338	0.0243	0.311	0.0226
B_11_	0.203	0.0152	0.197	0.0143	0.188	0.0132	0.197	0.0143
B_12_	0.211	0.0154	0.201	0.0146	0.193	0.0141	0.204	0.0148
B_13_	0.223	0.0161	0.204	0.0148	0.208	0.0149	0.209	0.0151
B_14_	0.279	0.0231	0.281	0.0229	0.289	0.0234	0.293	0.0237
B_21_	0.289	0.0239	0.292	0.0236	0.276	0.0225	0.299	0.0244
B_22_	0.256	0.0216	0.251	0.0207	0.288	0.0233	0.265	0.0212
B_23_	0.275	0.0227	0.267	0.0214	0.294	0.0236	0.272	0.022
B_24_	0.287	0.0238	0.277	0.0226	0.267	0.0219	0.284	0.0231
B_25_	0.245	0.0206	0.282	0.0229	0.288	0.0234	0.283	0.023
B_31_	0.164	0.0126	0.171	0.0124	0.188	0.0136	0.167	0.0119
B_32_	0.188	0.0138	0.182	0.0127	0.176	0.0126	0.193	0.0138
B_33_	0.199	0.0147	0.189	0.0138	0.179	0.0127	0.186	0.0135
B_41_	0.207	0.0151	0.234	0.0184	0.241	0.0201	0.253	0.0207
B_42_	0.255	0.0212	0.263	0.0208	0.257	0.0206	0.269	0.0211
B_43_	0.288	0.0239	0.291	0.0229	0.283	0.0228	0.274	0.0219
B_51_	0.244	0.0204	0.253	0.0207	0.252	0.0207	0.239	0.0197
B_52_	0.234	0.0201	0.229	0.0191	0.237	0.0189	0.267	0.0218
C_11_	0.198	0.0148	0.189	0.0137	0.179	0.0131	0.182	0.0134
C_12_	0.186	0.0142	0.181	0.0132	0.178	0.0130	0.174	0.0129
C_13_	0.165	0.0124	0.163	0.0117	0.168	0.0123	0.162	0.0118
C_21_	0.143	0.0108	0.146	0.0106	0.152	0.0109	0.151	0.0108
C_22_	0.154	0.0114	0.142	0.0102	0.148	0.0107	0.147	0.0104
C_23_	0.147	0.0113	0.149	0.0108	0.156	0.0112	0.149	0.0106
C_24_	0.125	00.097	0.123	0.0088	0.132	0.0094	0.133	0.0096
C_31_	0.162	0.0123	0.149	0.0107	0.148	0.0106	0.143	0.0103
C_32_	0.156	0.0117	0.161	0.0116	0.147	0.0104	0.157	0.0111
C_41_	0.101	0.0074	0.112	0.0081	0.108	0.0077	0.103	0.0072
C_42_	0.105	0.0077	0.102	0.0072	0.114	0.0081	0.107	0.0076
C_43_	0.122	0.0092	0.119	0.0086	0.113	0.0082	0.115	0.0083
D_11_	0.132	0.0096	0.129	0.0091	0.122	0.0088	0.115	0.0083
D_12_	0.175	0.0132	0.173	0.0124	0.179	0.0128	0.181	0.0129
D_13_	0.163	0.0122	0.164	0.0118	0.162	0.0117	0.163	0.0117
D_14_	0.154	0.0117	0.153	0.0112	0.155	0.0113	0.156	0.0113
D_21_	0.267	0.0223	0.278	0.0226	0.269	0.0219	0.271	0.0219
D_22_	0.298	0.0244	0.287	0.0231	0.283	0.0228	0.288	0.0232
D_23_	0.302	0.0246	0.299	0.0238	0.292	0.0234	0.296	0.0236
D_31_	0.205	0.0189	0.202	0.0181	0.208	0.0184	0.211	0.0187
D_32_	0.232	0.0197	0.243	0.0199	0.249	0.0204	0.245	0.0201
D_33_	0.223	0.0191	0.229	0.0191	0.232	0.0193	0.241	0.0196
E_11_	0.121	0.0091	0.124	0.0087	0.119	0.0087	0.129	0.0092
E_12_	0.124	0.0092	0.125	0.0088	0.122	0.0088	0.131	0.0093
E_21_	0.112	0.0083	0.106	0.0073	0.118	0.0086	0.109	0.0076
E_22_	0.113	0.0084	0.103	0.0072	0.115	0.0084	0.104	0.0073
E_31_	0.203	0.0183	0.211	0.0187	0.208	0.0183	0.201	0.0181
E_32_	0.243	0.0202	0.254	0.0208	0.247	0.0199	0.246	0.0198
E_41_	0.125	0.0093	0.119	0.0087	0.124	0.0088	0.122	0.0087
E_42_	0.102	0.0077	0.109	0.0077	0.113	0.0082	0.117	0.0084

By observing Table 2, the second level index of security risk of large-scale events such as music festival and football match are calculated and analyzed. The risk weight coefficient of human factors are about 0.3–0.4, the management and prevention factors are about 0.3–0.4, the site equipment are between 0.1 and 0.2, the event factors are about 0.2, and the audit factors are about 0.1. Among them, the human factors and management factors account for a large proportion, which is consistent with the design of human audit and management should be strengthened after major events were determined. The influencing factors in large-scale events are related to each other, which had obvious coupling relationship. The each assessment index should be paid the same attention due to each factor has certain risk.

In order to distinguish the security risk index system of important event, the objects assessment are carried out according to the criteria of excellent, good, medium and poor, and the corresponding objects closeness-degree are calculated according to formulas (12) and (13), as shown in Tables 3 and 4.

**Table 3:** Assessment criteria.

Objects	Excellent	Good	Medium	Bad
*Z*_*ij*_	0.0456	0.1732	0.2754	0.3214

**Table 4:** Assessment results.

Objects	P_1_	P_2_	P_3_	P_4_
*Z*_*ij*_	0.2631	0.2749	0.1531	0.2049
Assessment results	Medium	Medium	Good	Medium
Sort	3	4	1	2

Table 4 shows the security risk assessment closeness results and the comprehensive assessment results of the four events. The security risk assessment value of event P_3_ was 0.1531, which the assessment result is good. While the calculation results of P_1_、P_2_ and P_4_ closeness are between 0.01732 and 0.2754, and the assessment results are medium. Compared with each other, the four events are in a relatively security state. The assessment results are basically consistent with the security inspection results, which reflected the security level of the events process. The security degree of P_3_ and P_4_ events are better than P_1_ and P_2_ due to the participants in football events are more fixed and the change is relatively small, and events are carried out in the relative fixed field, which is conducive to security analysis and risk reduction. Music festivals are more complex, open and fewer security facilities. Among them, the lower risk of P_3_ is partly due to the Beijing government's higher requirements and stricter security audit. Before and after the events are carried out according to combine the assessment results with the index weight, the common problems such as the participant review, inspection of the equipment operation itself are payed attention, and the inspection safety management is strengthened to reduce the sudden accidents.

## Conclusion

The security risk index system and the assessment model with entropy weight method and material element model are constructed based on the causes of important events security accidents are analyzed. The subjectivity of the assessment process is reduced, the objective accuracy is improved, and the systematization of the assessment process is further optimized by the method. The practicability of the assessment model is verified by using examples.

Compared with the index system and entropy weight calculation results shows that: the weight of human factors and management factors in the process of holding important events are larger and more likely to induce security risks, so the construction of related systems such as personnel review and management should be strengthened. Because of the coupling of various factors, the awareness of security risk is strengthened, the supervision mechanism is improved, the ability of public security to deal with problems are strengthened, the quality of personnel and the level of technology are improved on the basis of effective assessment and improvement of hidden dangers of safety risks. So as to eliminate all kinds of security risks and hidden dangers.
